# Progress of the Medical Sciences

**Published:** 1914-03

**Authors:** 


### Progress of the Medical Sciences.

### MEDICINE.

**The Campaign against Tuberculosis.**—The *Transactions* of the Fifth Annual Tuberculosis Conference, held in August last at Westminster, contains much information as to the progress of the campaign against tuberculosis, and many papers in the *Journal of Tuberculosis* [1] for October deal with individual fragments of the work. The problems as they are being worked out in Wales and Monmouthshire are described by Dr. E. Walford, Cardiff. It is proposed that there shall be the following sanatoria: One for South Wales, providing for 250 adults and 50 children; one for North Wales, providing for 150 adults and 30 children; and one for West Wales, providing for 50 adults. There are to be thirteen hospitals for advanced cases. The King Edward Memorial Association is to provide two-fifths and the Treasury grant three-fifths of the capital expenditure. As regards the provision of sanatoria, Dr. Pearson's book [2] shows that "much is still only just being begun." He states the reasons which make it necessary for the State to provide and maintain sanatoria. He anticipates and apparently welcomes the gradual disappearance of efforts of voluntary charity, and their displacement by organised public relief. He considers that the majority of general practitioners apparently have not yet grasped the probable trend of events, that the number of thoroughly trained tuberculosis officers is admittedly too few, and that we should hear no more of the frequently repeated lament that there will be nothing left for the general practitioner to do. In the Government scheme the officials must each bear their part, the medical officer of health as co-ordinator and general administrative adviser, the tuberculosis officer filling the place of consultant diagnostician, examiner of contacts, and adviser as to the line of treatment to be adopted, while the general practitioner will chiefly be concerned in looking after the home conditions. As regards the Dispensary, its functions and work have been well summarised by Dr. King Patrick. [3] He claims that it must be the link binding the Public Health Department, the Sanatorium, the Hospital for Advanced Cases, the Labour Colony, the School Medical Inspection Department, voluntary agencies, and the general practitioner, to one another, and correlate the work of each. In the carrying out of all these details, Dr. Pearson remarks that "those who already have an experience of managing sanatoria, dispensaries, and other institutions for tuberculosis, sometimes complain that the Councils are unsympathetic and untutored." [4]

The advent of the tuberculosis scheme of the National Insurance Act is a fitting time for the appearance of a new annual[5] dealing comprehensively with the whole subject. Science and philanthropy have done much good work in the development of the matured campaign of the national and international aspects of the war against tuberculosis, and now that the Governments are taking up the work, the co-operation of the existing authorities must be of the utmost value in working on the already proposed basis. The year-book compiled under the editorship of Dr. Kelynack aims at being something more than a directory of sanatoria, hospitals, homes and other institutions for the tuberculous. It seeks to provide a reliable guide to all matters relating to the study and care of the tuberculous. A series of original articles by well-known experts have been well selected, and gives the latest views on all the many questions relating to sanatoria and sanatorium manage, ment: the "Editorial Review" is a valuable communication showing how much has been already accomplished, and the leading ideas for future progress. We are now in a position to determine whether things are progressing in a satisfactory direction, or whether endeavours should not be made to amend certain features of the programme.

**The Tuberculosis Officer.**—As regards the duties of this new officer, these have been more or less defined by orders of the Local Government Board, and have been emphasised by the Astor Report:[6] "He will act in intimate relationship with the medical officer of health, the general practitioner, and the resident medical officers of sanatoria and hospitals, but in his clinical capacity he will be independent of control. At the dispensary his clinical duties will be chiefly concerned with diagnosis and treatment, and he will be called upon to decide as to the suitability of various types of tuberculous disease for dispensary, domiciliary, hospital, and sanatorium treatment." It will be seen that his duties call for some administrative experience, sound clinical knowledge, with much tact and discretion. He must, therefore, be not too young, he must realise his limitations in doubtful cases, and be ready to co-operate with his colleagues in clearing up any doubts which may arise either as regards diagnosis or treatment.

**The Sanatorium Medical Staff.**—The Sanatorium Superintendent must be somewhat of a tyrannical autocrat, and he has been well described by F. M. Pottenger, of the California Southern University: "A sanatorium is simply the reflection of the man who is at the head of it, and whether it succeeds or fails depends upon him. The sanatorium treatment of tuberculosis requires of a medical director certain important qualifications. He should first of all understand the disease which he is expected to treat, and all things being equal, the better he understands it the better will be his results. Next, and not of less importance, he must be possessed of patience. He must be sympathetic, yet firm. He must be optimistic, but his optimism must be bounded by reason. He must be endowed with qualities of leadership, for he must not only lead his patients, but he must command an army of help. Sanatorium men are born, not made. It is useless to think that a sanatorium can be properly conducted by simply placing a physician, regardless of his qualifications and temperament, at the head of it. The head of a sanatorium should live at the institution, so that he may come into intimate contact with his patients, and be in touch with everything that goes on. The medical director of a sanatorium occupies much the same position as the captain of the ship. Harmony must be everywhere preserved, therefore every department should be under his direction and personal supervision.

"A word regarding medical directors for State sanatoria. These institutions should not be controlled by politics . . . their success depends upon the men at the head of them. It can readily be seen that one fit for such a position is fit to succeed anywhere. Such a position should carry with it a good salary. . . . Unless the board of directors of State institutions look well to this point they will be disappointed in the results obtained."

The private sanatoria for the better class patient who can afford to pay adequately for his maintenance have been in existence for several years, and have been looked upon as models on which such institutions should be framed, but now the general outcry is that these are too expensive, and that a cheaper form of sanatorium, with a less abundant medical staff, becomes essential. Many such exist, and the tendency is to multiply beds unduly, until the patients are too numerous to receive adequate individual attention from the existing staff. One institution familiar to the writer at the present time has 86 beds in constant occupation, with only one medical officer and no visiting physicians. This medical officer has practically no assistance in the daily clinical routine, nor in the registration of this large number of patients. He is also expected to carry out the pathological examinations and the dispensing as well. The position was found to be so untenable, that the last medical officer resigned his office rather than continue to perform the work inefficiently and inadequately.

This question of adequate medical superintendence is one which demands the most careful consideration, and one which is not understood by the lay authorities. Members of Insurance and other committees appear to think that medical supervision is for the most part a mere matter of routine, and that the number of patients may be increased almost indefinitely without increasing the medical staff. The contrast between the private and the more public sanatoria in this respect is great, as, for instance, one institution known to the writer where thirty-seven patients have two medical officers and a medical assistant, whereas at the other extreme we have eighty-five patients with only one medical officer, who does everything. Now it is well recognised by all who have had experience of this disease that no two patients can be classified as alike, and they must, therefore, receive individual attention and careful consideration from day to day. One medical sanatorium officer writes: "Personally, I have always found that forty chests are ample to have in my mind at once." When the number of patients at one time is greatly in excess of this number, it is impossible that the medical officer shall give such attention as is likely to be effective. As the number increases, the quality of the work must deteriorate, and it will generally be held by medical men that ineffective work must not be tolerated.

Pearson[7] remarks that "when the State undertakes the provision of sanatorium treatment for tuberculosis, the amount of personal attention required from the doctor of the institution must be ample. What is considered ample at present may soon be thought to be inadequate. Sanatoriums of 150 to 250 beds are now contemplated, and are already in existence. For such an institution three (or four) doctors are considered sufficient. But the essence of recent advances in medical treatment is the appreciation of the necessity for close personal supervision. . . . I believe, therefore, that a good arrangement to aim at in the management of State sanatoriums is that of having an institution where the maximum number of patients is 75. Then one head doctor with two assistants can study sufficiently closely all the patients under his care."

Walters[8] gives some useful remarks on the medical staff. The number of patients which can be properly supervised by each medical officer depends largely upon their condition. Not more than thirty or forty patients should be allotted to each doctor if they are mainly febrile or complicated cases; but double this number of ambulant uncomplicated cases could quite well be manged. Since one medical officer at least must always be in the sanatorium, every such institution should have not less than two medical efficers, otherwise the strain of the work becomes very irksome.

Every large sanatorium requires a competent pathologist to attend to the microscopical and bacteriological work, which will include the preparations of vaccines and of doses of tuberculin.

In the early days of sanatorium work it has been impossible to provide for more than one medical officer at a new institution to be maintained by public and philanthropic efforts, but this arrangement has many disadvantages and difficulties. It would be better to have two medical officers with a larger number of beds than one doctor with a small number of patients, for many reasons. There should, of course, be one medical officer always on the premises, especially when the institution is away in a country district, with no other medical aid at hand in case of necessity. Then again, one of two medical officers could be present at and supervise the principal meals; and it would be possible for one of two to superintend graduated exercises and labour in a way which must be impossible with only one. For these and many other reasons it is obvious that the institution with only one medical officer should be a thing of the past, and now that State aid is becoming general, we should no longer hear of any sanatoria where the one doctor has no regular hours off duty, and little opportunity of getting away from duty at any time. The isolated life and the sole responsibility which have existed hitherto should no longer be countenanced. Consultation with a colleague and division of labour with some degree of specialisation, as for instance the dealing with dental, oral, laryngeal and nasal cases, must often be desirable, and should be possible at all times.

**Drug Treatment of Pulmonary Tuberculosis.**—It is commonly assumed that the sanatorium takes the place of drug treatment, and that there is no need for the latter, but it is gratifying to find an excellent article by Dr. Newman Neild,[9] from which we quote as follows: "Under the sanatorium method it was important to make the patient 'concentrate' upon the system, so that full attention to and full reliance upon 'playing the game' be not disturbed by belief in a bottle of medicine. Moreover, since certain symptoms, such as night-sweats and insomnia, generally disappear under sanatorium treatment in a few days, the digestion improves, and cough becomes less troublesome, drugs took up a less important place. Formerly treatment was too symptomatic, and recently the symptoms have been too much neglected. . . . Many have wondered why drugs, which were valuable aids under less hygienic conditions, became useless under better conditions. . . . That 'cures' are still in the minority under tuberculin we must admit with regret."

There seems to be still a wide field for drugs, and an excellent summary of what drugs can do is found in this article. The use of continuous antiseptic inhalations is described by Dr. David B.

Lees (p. 148), and is also mentioned by Dr. C. Muthu (p. 252), whose various papers on the subject are well known.

R. SHINGLETON SMITH.

REFERENCES.

[1] 1913, vii, 19.

[2] *The State Provision of Sanatoriums.* S. V. Pearson, M.D. Cantab., 1913.

[3] *Brit. J. Tuberculosis,* 1913, vii. 228.

[4] *Ibid.,* 1914, viii. 10.

[5] *Tuberculosis Year-Book and Sanatoria Annual.* T. N. Kelynack, M.D. Vol. i, 1913–14. London: John Bale, Sons & Danielsson.

[6] *Interim Report of Dept. Committee on Tuberculosis.* London: Wyman & Sons. 1912.

[7] *State Provision of Sanatoriums,* 1913, p. 48.

[8] *Sanatoria for the Tuberculous.* F. R. Walters. London: Geo. Allen & Co. 1913.

[9] *Year-Book,* p. 142.

### SURGERY.

**Embolism and Thrombosis of the Mesenteric Vessels.**—Of the various causes of acute abdominal trouble the above vascular accidents are among the most difficult to recognise and treat. An excellent and comprehensive review of the subject, based on the records of 360 cases, seven of them hitherto unpublished. has recently been written by Leslie B. C. Trotter, [1] and it is from that publication that the information given below has been abstracted.

*Historical.*—The first case of the kind recorded appears to be that of Tiedman in 1843, and the next cases were three described by Virchow in 1847 and 1854. Trotter himself has examined the records of 360 cases, as mentioned above, a fact which well illustrates the increasing attention which is being given to the subject.

*Morbid Anatomy.*—Mesenteric vascular occlusions may be of veins alone, of arteries alone, or of both arteries and veins simultaneously. The clinical phenomena associated with these various occlusions are practically never clear enough for their differential diagnosis. Of Trotter's cases about 41 per cent. were of venous, 53 per cent. of arterial, and 6 per cent. of combined venous and arterial obstruction.

*Mesenteric Venous Occlusion.*—Occlusion of the inferior mesenteric vein is clinically unimportant. Probably occlusion of the superior vein leads to infarction of the intestine only when

[1] *Embolism and Thrombosis of the Mesenteric Vessels.* By Leslie B. C. Trotter, M.A., B.C. Cantab.—Cambridge University Press, 1913.

the clot extends into the anastomising arcades, and their radicles, close to the intestine. The thrombosis may be primary in the superior mesenteric vein, or secondary to a descending thrombosis of the portal vein. The latter is the commoner. All the possible causes of pressure upon the portal vein, *e.g.* syphilitic perihepatitis, alcoholic cirrhosis or carcinoma of the liver, glands or other swellings in the portal fissure, gall stones, etc., by obstructing the circulation, are predisposing causes of thrombosis. When sepsis is superadded to portal obstruction, then if thrombosis occurs its peripheral extension becomes likely. Thus sepsis in any part of the alimentary tract draining into the portal system may cause thrombosis. Primary thrombosis of the superior mesenteric vein may arise in a similar way. The commonest septic foci having these effects are those in connection with the appendix, and in the female with the pelvic organs. Of the 137 cases of venous thrombosis of Trotter's collection, there were 91 in which the intestine showed infarction.

*Arterial Thrombosis and Embolism.*—Of 197 cases of these affections in the series, 106 showed infarction of the intestine, 36 hemorrhages or great engorgement, and 10 ulceration of the mucosa. The percentage of serious effects is thus a high one in arterial obstruction. This is the case because the superior mesenteric artery is "functionally," though not anatomically, an end artery. There are ten cases on record in which collateral circulation was established before death, but in six of these it proved ultimately insufficient. The arteries may be occluded by thrombosis or embolism, or more rarely by endarteritis, arterio-sclerosis, or atheroma either of the arteries themselves or in the aorta at the place of their origin. By many authorities arterio-sclerosis of the mesenteric vessels is considered to be a very important etiological factor in thrombosis. The sclerosis may give rise to a prodromal clinical condition, "intermittent mesenteric claudication," in which there are attacks of spasmodic pain in the bowels and other symptoms of disorder and motor insufficiency, attributed to local anæmia, "where greater performance of work is required of the intestine." Embolism of the mesenteric arteries is much commoner than thrombosis. The embolus comes from the pulmonary vessels, the heart, or the aorta; by far most frequently from the heart. "There are only two undoubted cases of embolism of the inferior mesenteric artery alone." It is very rare also to have the superior and inferior arteries simultaneously occluded. There is a considerable amount of evidence supporting the view that the intestinal and other lesions found in Henoch's purpura are of embolic origin, the emboli coming from the heart. Some cases of mesenteric embolism have been associated with purpuric hemorrhages. Simultaneous occlusion of mesenteric arteries and veins has only been recorded in about twenty-two cases.

*Effects on the Intestine.*—The effects of occlusion of the vessels are (1) hemorrhagic infarction, or less severe hemorrhagic changes, and (2) anæmic gangrene or ischæmia of the intestine. The extent of the infarction varies. There may be merely a few necrotic areas, or a short coil of intestine may be involved ; or, on the other hand, the whole of the large and small intestines may be affected. Rarely two or more separated infarctions occur.

Anæmic gangrene, of which Trotter tabulates data of nine cases, has not been satisfactorily explained. The obstruction seems in the cases of this type to have been in the vessels close to the intestinal walls, and was possibly preceded, as Trotter suggests, by a steady reduction in the size of the vessels supplying the part, affecting the veins as well as the arteries, so that when complete obstruction ensued there was no venous regurgitation.

*Symptomatology.*—The great majority of cases run their course in fourteen days, and many in a much shorter period of time. The division made by Trotter into acute cases lasting a week or under and chronic cases lasting eight days or more is arbitrary, but has conveniences. A greater proportion of arterial than venous cases are acute. The disease has diverse clinical forms, and it is rare for a given case to present more than a few of the symptoms enumerated below. Though the onset is usually acute, yet conditions predisposing to the affection may have been recognisable months or years previously. Such conditions include valvular heart disease, hepatic cirrhosis and chronic Bright's disease. A striking feature of the cases arising from appendicitis or pelvic septic processes is a latent period varying from a few days' duration to six or eight weeks.

The following are the characteristic symptoms and signs which have been met with in the recorded cases. Sudden severe abdominal pain, nausea, vomiting of blood-stained matter, diarrhœa with blood in the evacuations, collapse with anxious expression and clammy skin, and rapid pulse. The temperature may be raised, normal, or sub-normal. The abdomen early becomes tender. Constipation, often absolute, follows diarrhœa, though sometimes the bowels act normally until constipation ensues. Pain may abate for a day or two, and then increase again, with continuous vomiting of dark-coloured and offensive matter. Finally the patient often becomes comatose. Possibly the absence of intestinal obstruction observed in some cases was due to the fact that the patient did not survive long enough for this effect of the disease to become prominent. The amount of blood in the stools may be small or great in the earlier stages, and sometimes there is a discharge of blood shortly before death, following the period of intestinal obstruction. In the diarrhœa stage rectal tenesmus is not uncommon, and the motions may be thin, watery and offensive. Hiccough, probably from the onset of peritonitis, may be a source of great distress. In a few cases an ill-defined mass has been felt in the abdomen, due to swelling of the mesentery and intestinal walls. Respirations tend to become thoracic in type, and œdema of the bases of the lungs may follow.

*Diagnosis.*—This is extremely difficult, and was only made thirteen times in Trotter's series of 360 cases. One cause of error in diagnosis is the variability and incompleteness of the clinical picture, and another the fact that hitherto the disease has been considered too rare to merit attention. The existence of endocarditis and evidence of embolism elsewhere would suggest the correct diagnosis in embolic cases. Perhaps the distinction from *acute intussusception* is most difficult. The age of the patient affords some help, for mesenteric obstruction, though it may occur at any age, and has been recorded at the age of a month at the one extreme and at 85 at the other, yet is much more prone to occur between the ages of 30 and 70. The presence of a sausage-shaped tumour would strongly point to intussusception. In *volvulus* there will usually be more complete intestinal obstruction from the beginning, and the abdominal distention will be more rapid and greater. In *carcinoma of the colon* there may be a suggestive previous history, and visible peristalsis is likely to be present. The latter is extremely rare in mesenteric thrombosis and embolism. In *internal strangulation* hæmatemesis is very rare, and the onset of intestinal obstruction is usually more sudden. The complications which may follow appendicitis with acute intestinal obstruction are very difficult to differentiate from one another. They include toxæmia, peritonitis, adhesions and infarction. The appearance of blood in the stools, after or without an enema, would be helpful in differentiating *acute pancreatitis.*

*Treatment.*—Operative exploration in all severe cases at an early period is strongly indicated, for the termination of the disease is nearly always fatal if large or medium-sized vessels are obstructed, and relief is not obtained by radical procedures. Resection of the infarcted portion of the bowel is the ideal treatment. Occasionally affected intestine may be found on exploration which is still viable, and recovery take place without resection. "The results of operation, exclusive of cases in which nothing was attempted, give 36.2 per cent. of recoveries" (Trotter). It is not considered prudent to remove more than one-third of the total length of the small intestine. If less than a certain amount is left inanition follows, with intractable diarrhœa. Gobiet resected 320 cms. with success. Trotter quotes a paper by Flint[1] on the subject of extensive resections of the bowel. Flint collected fifty-nine cases in which such resections had been made. In all but one case two metres (78.74 inches) and over were removed, and fifty of the fifty-nine patients recovered.

J. LACY FIRTH.

### Reviews of Books.

[1] *Johns Hopkins Hosp. Bull.*, 1912, xxiii, 127.

